# Interface engineering V_2_O_5_@PANI nanotube for high-performance aqueous zinc-ion batteries

**DOI:** 10.1016/j.isci.2025.114337

**Published:** 2025-12-04

**Authors:** Shen Wang, Xueying Sun, Hongbo Xu

**Affiliations:** 1 College of Chemical and Material Engineering, Quzhou University, Quzhou 324000, China; 2 MIIT Key Laboratory of Critical Materials Technology for New Energy Conversion and Storage, School of Chemistry and Chemical Engineering, Harbin Institute of Technology, Harbin 150001, China

**Keywords:** Energy engineering, Energy systems, Energy storage

## Abstract

Aqueous Zn-ion batteries offer safe and cost-effective energy storage, yet their energy density requires improvement. While vanadium pentoxide (V_2_O_5_) possesses attractive redox activity, its practical implementation is hindered by structural instability and limited cycling performance. Core-shell V_2_O_5_@PANI nanotubes are fabricated via *in situ* polymerization to enhance Zn^2+^ diffusion and structural resilience. This architecture facilitates interfacial charge transfer and stabilizes the host framework during cycling. The optimized cathode achieves a high capacity of 462.4 mAh g^−1^ at 0.5 A g^−1^ with 96.5% capacity retention over 500 cycles. This work demonstrates a synergistic combination of nanostructural control and interfacial engineering, providing a generalizable materials design strategy for advanced electrochemical energy storage systems.

## Highlights

• Core-shell V₂O₅@PANI nanotube fabricated via *in situ* oxidative polymerization
• PANI coating expands interlayer spacing, enhances conductivity, and stabilizes framework
• Composite delivers 462.4 mAh g^−1^ capacity with 96.5% retention over 500 cycles

## Graphical abstract

**Figure:**
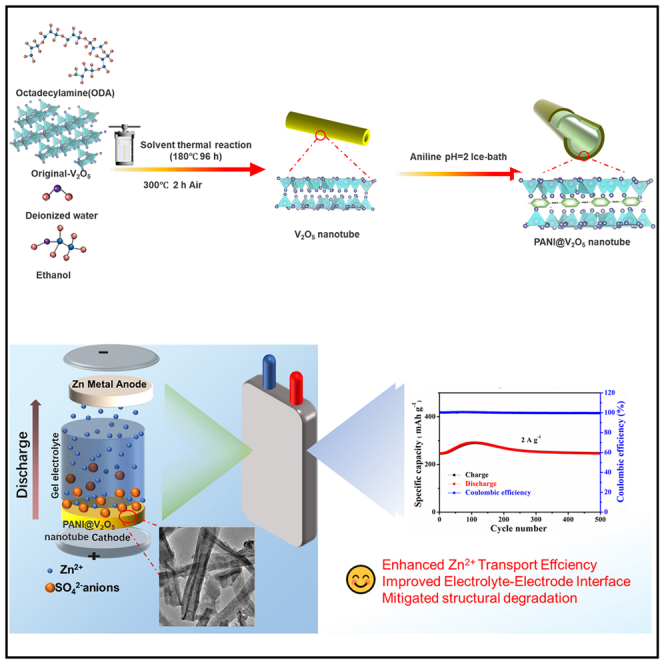

## Introduction

Aqueous zinc-ion batteries (AZIBs) have emerged as a highly promising candidate for large-scale electrochemical energy storage, benefiting from inherent operational safety and cost-effectiveness.^1^^,^^2^^,^^3^ Among various cathode materials, vanadium pentoxide (V_2_O_5_)-based compounds stand out due to high theoretical capacity (589 mAh g^−1^), broad voltage window, improved safety, and low cost.^4^^,^^5^^,^^6^^,^^7^^,^^8^ However, their practical application is hindered by intrinsic limitations such as narrow interlayer spacing, poor electronic/ionic conductivity, and structural degradation during cycling.^9^^,^^10^^,^^11^^,^^12^

To overcome these challenges, several modification strategies have been explored. One approach focuses on nanostructuring V_2_O_5_ into forms such as nanopaper,^13^ nanosheets,^14^ or nanotubes,^15^ which increases the specific surface area and promotes electrolyte accessibility, thereby accelerating redox kinetics. Another method involves pre-intercalating metal ions (e.g., Co^2+^, Ca^2+^, and Fe^3+^), which serve as structural pillars to widen the interlayer spacing and stabilize the host framework.^16^^,^^17^^,^^18^^,^^19^ A third route entails constructing V_2_O_5_ composites with conductive polymers (e.g., polyaniline [PANI], polypyrrole, poly(3,4-ethylenedioxythiophene)) or molecular modifiers (e.g., H_2_O, organic dyes), which enhance Zn^2+^ storage and cycling performance.^4^^,^^6^^,^^10^^,^^20^^,^^21^^,^^22^^,^^23^^,^^24^ While these intercalants expand the interlayer distance and improve rate capability, their small molecular dimensions often lead to electrochemical dissolution during repeated cycling, resulting in gradual structural decay.^25^^,^^26^

In this context, PANI presents a compelling alternative as a pillaring agent, owing to its structural robustness, tunable chain dimensions, and intrinsic Zn^2+^ storage capability.^22^^,^^26^^,^^27^^,^^28^^,^^29^^,^^30^ The strategic insertion of PANI into V_2_O_5_ interlayers not only expands the intergalley spacing but also mitigates interlayer electrostatic repulsion, thereby reinforcing the host matrix. Despite these advantages, the integration of PANI with well-defined one-dimensional V_2_O_5_ nanotubes to form a core-shell heterostructure remains unexplored for AZIB cathodes.

Herein, we report for the first time the rational design of a V_2_O_5_@PANI core-shell nanotube composite (denoted as V_2_O_5_@PANI-NT) through *in situ* oxidative polymerization of aniline on solvothermally derived V_2_O_5_ nanotubes. The novelty of this architecture lies in its synergistic combination of nanotube morphological and interfacial engineering: (1) the nanotube framework accommodates volume variation and provides mechanical stability during cycling; (2) PANI intercalation expands the interlayer channels, facilitating rapid Zn^2+^ diffusion; and (3) the conformal PANI coating enhances electronic conductivity and suppresses structural degradation. As a result, the V_2_O_5_@PANI-NT-based cell demonstrates remarkable specific capacity, high energy density, and extended cycling life. This integrated strategy offers a new pathway for developing high-performance V_2_O_5_-based cathodes for AZIBs, moving beyond conventional single-parameter modifications.

## Results

### Chemical composition analysis

**Scheme 1:**
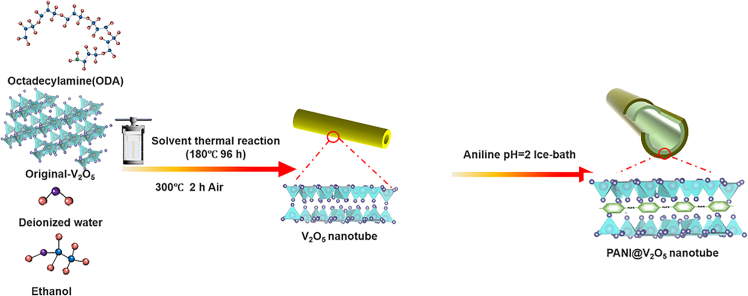
Schematic illustration for the synthesis of PANI@V_2_O_5_ nanotube

**Figure 1:**
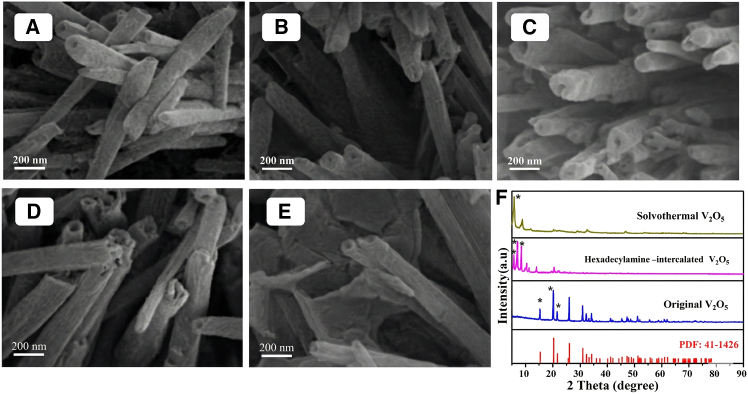
SEM image of V_2_O_5_-NT with different molar ratios of 1-hexadecylamine to V_2_O_5_ (A) V_2_O_5_-NT-1/2. Scale bars, 200 nm. (B) V_2_O_5_-NT-3/4. Scale bars, 200 nm. (C) V_2_O_5_-NT-1/1. Scale bars, 200 nm. (D) V_2_O_5_-NT-4/3. Scale bars, 200 nm. (E) V_2_O_5_-NT-2/1. Scale bars, 200 nm. (F) XRD patterns of V_2_O_5_ at different states.

The fabrication of high-quality V_2_O_5_@PANI-NT composites is fundamentally dependent on the synthesis of well-defined V_2_O_5_ nanotubes. As outlined in Scheme 1, V_2_O_5_-NT was prepared by dissolving V_2_O_5_ and 1-hexadecylamine in a mixed solvent of H_2_O and ethanol, followed by solvothermal treatment at 180 °C. To optimize the structural properties of V_2_O_5_-NT, key synthesis parameters—including solvothermal duration, annealing temperature, and the molar ratio of 1-hexadecylamine to V_2_O_5_—were systematically investigated. The corresponding results are summarized in Figures 1 and S1–S6.

Scanning electron microscopy (SEM) images of the precursor material reveal a well-defined lamellar morphology with distinct two-dimensional characteristics (Figures S1A and S1B). Upon extending the solvothermal treatment to 48 h, a notable morphological evolution occurs, in which portions of the nanosheets begin to curl and initiate nanotube formation (Figure S1C). Further prolonging the reaction to 72 and 96 h results in samples composed predominantly of nanotubes, with the most well-defined and uniform architecture achieved after 96 h (Figures S1D and S1E). Complementary X-ray diffraction (XRD) analysis of samples obtained at different reaction stages (Figure S2) provides insight into the accompanying crystal structure evolution and further clarifies the role of 1-hexadecylamine in directing the morphological transformation. Based on the time-dependent structural development observed by SEM, a solvothermal duration of 96 h was identified as optimal for the formation of high-quality V_2_O_5_ nanotubes and was accordingly adopted in all subsequent experiments.

Open-ended V_2_O_5_ nanotubes were synthesized via a solvothermal method using 1-hexadecylamine as a structure-directing agent with a reaction duration of 96 h. To systematically examine the influence of the 1-hexadecylamine-to-V_2_O_5_ molar ratio on the resulting morphology, a series of V_2_O_5_-NT-z samples were prepared with varying molar ratios (z = 2:1, 4:3, 1:1, 3:4, and 1:2). During nanotube formation, 1-hexadecylamine fulfills a dual function: it intercalates into the V_2_O_5_ interlayers to expand the interlamellar spacing, while also serving as a soft template that promotes layer exfoliation and curling. Consequently, its concentration directly governs the wall thickness of the resulting nanotubes. SEM images (Figures 1A–1E) clearly demonstrate that as the proportion of 1-hexadecylamine increases, the nanotube wall thickness progressively decreases. A molar ratio of 1:1 yields nanotubes with moderate wall thickness and well-defined tubular morphology. In contrast, at a ratio of 2:1, the V_2_O_5_ layers become excessively thin and fail to accumulate adequate curling stress to form intact nanotube structures. Based on these morphological and structural analyses, a 1:1 molar ratio of 1-hexadecylamine to V_2_O_5_ and a reaction time of 96 h were selected as the optimal conditions for all subsequent preparations of V_2_O_5_-NT.

To elucidate the formation mechanism of the nanotubes, intermediates isolated at different stages of the process were characterized by XRD and SEM (Figures 1F and S4). Integrating these results, the structural evolution can be summarized as follows: pristine V_2_O_5_ nanosheets are first intercalated by 1-hexadecylamine, leading to expanded interlayer spacing. The intercalated sheets then undergo edge-initiated self-curling driven by accumulated interfacial stress, followed by gradual fusion into continuous, hollow nanotube structures. A schematic representation of this proposed formation pathway is provided in Figure S4, visually summarizing the transformation from layered precursors to final V_2_O_5_-NT.

It should be noted that the as-synthesized V_2_O_5_-NT samples retain residual organic species, such as 1-hexadecylamine, which remain embedded within the nanotube framework. These residues can act as insulating barriers, impeding ion transport and reducing the accessibility of electroactive sites, thereby adversely affecting the capacity and rate performance in energy storage applications. Therefore, a subsequent calcination step is essential to remove organic residues and optimize the electrochemical properties of the V_2_O_5_-NT material.

The influence of calcination temperature on the structure and chemical evolution of V_2_O_5_ nanotube was systematically investigated via SEM, XRD, high-resolution transmission electron microscopy (HRTEM), and X-ray photoelectron spectroscopy (XPS). As illustrated in Figure S4, samples annealed at 200°C, 300°C, 400°C, and 500°C reveal a clear thermal threshold: organic residues persist below 300°C, while higher temperatures induce nanotube collapse. Consequently, 300°C was established as the optimal annealing condition, achieving complete removal of organics while retaining structural integrity. HRTEM results (Figure S5) further revealed that annealing at 300 °C induces a structural contraction, reducing the interlayer spacing from 2.14 to 0.98 nm. This lattice densification stems from the removal of intercalated 1-hexadecylamine, which diminishes interlayer support and promotes structural reorganization, thereby enhancing the mechanical stability of the V_2_O_5_ nanotube framework. XPS analysis (Figure S6) offers further insight into the chemical evolution after 300°C treatment, revealing a substantial increase in V^5+^ content. This shift results from the elimination of the reducing amine environment and concomitant oxidation of V^4+^ species. The elevated V^5+^ concentration significantly improves electrochemical behavior: its enhanced redox activity not only offers more electron-transfer sites but also promotes the *in situ* oxidative polymerization of aniline in subsequent processing. Moreover, the stronger electrostatic attraction associated with V^5+^ facilitates Zn^2+^ intercalation and improves the structural stability of the host framework. Therefore, annealing at 300°C optimally balances the removal of organic templates with the preservation of nanotube morphology, while also inducing favorable structural contraction and elevating the vanadium oxidation state. These synergistic effects collectively enhance the electrochemical performance of V_2_O_5_-NT, validating its use as an advanced precursor for the construction of V_2_O_5_@PANI-NT composites.

As supported by previous reports,^26^^,^^27^^,^^28^^,^^29^^,^^30^ the redox potential of V_2_O_5_ under acidic conditions is sufficiently high to drive the oxidative polymerization of aniline. In this work, pre-synthesized V_2_O_5_ nanotubes served a dual role: as an oxidizing agent to initiate polymerization and as a structural scaffold to guide the formation of a core-shell V_2_O_5_@PANI-NT composite.

Systematic investigation of the reaction pH revealed its critical influence on both morphology and reaction kinetics. As shown in Figure S7, under strongly acidic conditions (pH ≤ 1.5), the nanotube structure was severely etched and degraded. In contrast, at higher pH values, the oxidizing capability of V_2_O_5_ was notably suppressed, leading to sluggish polymerization kinetics and incomplete PANI formation. Therefore, a pH of 2 was identified as the optimal trade-off, enabling efficient aniline polymerization, while preserving the structural integrity of the V_2_O_5_ nanotube template.

Furthermore, a reaction time of 2 h was found to be optimal for constructing a well-defined core-shell architecture with maintained nanotube morphology (Figure S8). The resulting V_2_O_5_@PANI-NT composite is expected to exhibit enhanced electrical conductivity, expanded interlayer spacing, and improved Zn^2+^ storage capacity, while retaining the underlying V_2_O_5_ framework. XRD analysis (Figure S9) provided additional structural evidence: the decreased diffraction intensity indicated increased amorphicity due to PANI coating, while the shift of the (001) peak to lower angles confirmed the expansion of interlayer spacing, consistent with successful PANI intercalation.

**Figure 2:**
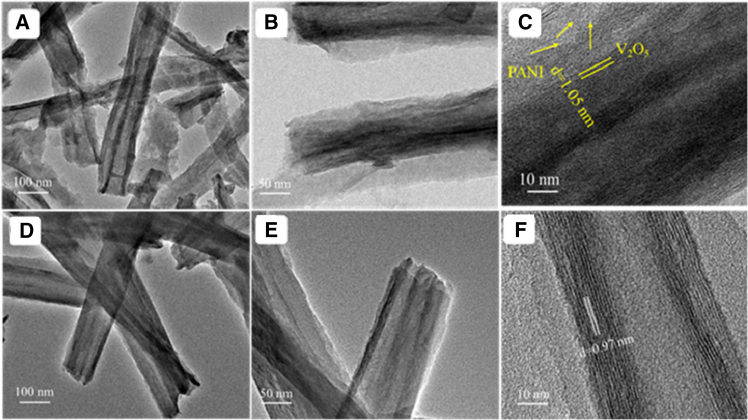
TEM and HRTEM image of V_2_O_5_@PANI-NT and V_2_O_5_-NT (A–C) V_2_O_5_@PANI-NT at different magnifications. Scale bars, 100 nm in (A), 50 nm in (B), and 10 nm in (C). (D–F) V_2_O_5_-NT at corresponding magnifications. Scale bars, 100 nm in (D), 100 nm in (E), 10 nm in (F).

As depicted in Figure 2, the V_2_O_5_@PANI composite preserves the original nanotube architecture while forming a well-defined core-shell structure, where a V_2_O_5_ core is uniformly encapsulated by a conformal PANI shell. HRTEM analysis confirms that these composite nanotubes have a narrow size distribution, with diameters primarily ranging from 100 to 120 nm. A key structural change is the expansion of the (001) interplanar spacing from 0.97 nm in pristine V_2_O_5_-NT to approximately 1.05 nm in the composite. This discernible lattice expansion provides direct evidence for the successful intercalation of PANI molecules into the V_2_O_5_ interlayers. Such structural evolution not only confirms effective interfacial interaction but also underpins the enhanced ion diffusion and structural stability of the composite.

Raman and Fourier transform infrared spectroscopy (FTIR) analyses (Figure S10) provide definitive evidence for the PANI cladding and reveal the nature of the interface in the V_2_O_5_@PANI-NT composite. The Raman spectrum (Figure S10A) reveals the coexistence of V_2_O_5_-NT features and characteristic PANI signatures, including benzene ring vibrations (1,100–1,700 cm^−1^) and a C–N stretching mode at ∼1,339 cm^−1^. A critical observation is the red-shift of the V–O vibrational peaks, indicating a decreased bond order resulting from electron density redistribution at the interface, likely driven by Lewis acid-base interaction between PANI (N) and V_2_O_5_ (O). The FTIR spectrum (Figure S10B) further corroborates this redox-mediated interfacial chemistry: the emergence of PANI-specific absorption bands coincides with spectroscopic features indicating the reduction of V^5+^ to V^4+^, confirming that V_2_O_5_ acts as an oxidant for aniline polymerization. This mechanism is consistent with the distinct weight loss step observed in the thermogravimetric (TG) profile (Figure S11) between 200°C and 400°C, assigned to PANI decomposition. The combined data unequivocally confirm the successful integration of PANI via a robust interfacial bond, creating a functional heterostructure that enhances functionality, while preserving the architectural integrity of the V_2_O_5_ nanotube host.

### Electrochemical performance

**Figure 3:**
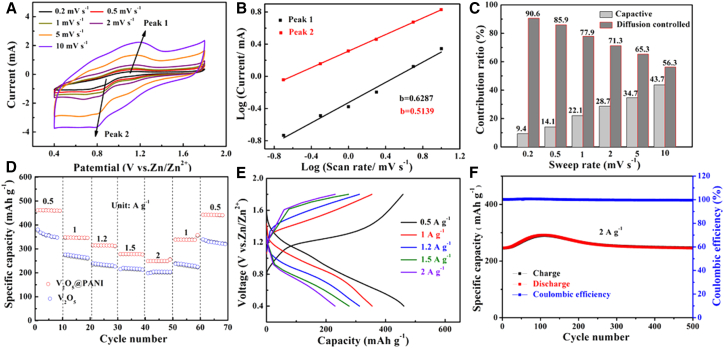
Electrochemical properties of PANI@V_2_O_5_-NT//Zn battery (A) CV curves of the battery at different scan rates. (B) Log(i) versus log(*v*) plots at specific peak currents. (C) Contribution ratios of capacitance control and diffusion control. (D) Rate performance of the cell at various current densities. (E) Charge-discharge curves of the cell at different current densities. (F) Cyclic performance and coulombic efficiency of the cathodes at 2.0 A g^−1^.

To systematically evaluate the electrochemical performance, a quasi-solid-state zinc-ion battery was assembled employing the as-prepared material as the cathode, a Zn mesh as the anode, and a ZnSO_4_/polyvinyl alcohol (PVA) gel as the electrolyte. The electrochemical behavior was first probed by cyclic voltammetry (CV). As shown in Figure 3A, the CV curves of the V_2_O_5_@PANI-NT//Zn cell, measured at scan rates from 0.2 to 10.0 mV s^−1^ within a voltage window of 0.4–1.8 V, consistently exhibit two distinct pairs of redox peaks. These well-defined peaks correspond to a two-step Zn^2+^ insertion/extraction process, and their highly symmetric shape is indicative of highly reversible faradaic reactions, which is characteristic of battery-type electrode materials. Furthermore, the current response increased progressively with the scan rate, while the CV profiles remained largely unchanged, demonstrating excellent reaction kinetics and structural stability.

To gain deeper insights into the charge storage mechanism, we performed quantitative kinetic analysis. Based on the relationship between peak current (i) and scan rate (*v*) (see Equations 1 and 2), the calculated b-values of approximately 0.63 for Peak 1 and 0.51 for Peak 2 suggest a mixed charge storage mechanism involving both diffusion-controlled and capacitive processes (Figure 3B). This intermediate b-value indicates that the electrochemical process is neither purely diffusion limited nor surface controlled, but rather a combination of both mechanisms, which is advantageous for achieving both high capacity and good rate performance.

Further analysis using the Dunn method (Equation 3) reveals that at a scan rate of 0.2 mV s^−1^, the pseudocapacitive contribution accounts for 9.4% of the total capacity, increasing progressively to 47.3% at higher scan rates (Figure 3C). This behavior can be understood by considering the unique architecture of our material: the nanotube morphology provides abundant surface sites for fast capacitive storage, while the expanded interlayer spacing through PANI intercalation maintains efficient diffusion pathways for bulk redox reactions. Despite the substantial increase in capacitive contribution at high rates, the diffusion-controlled process remains dominant across all scan rates (exceeding 50%), with a markedly greater contribution compared to pure V_2_O_5_-NT (Figure S12), indicating that the material preserves its battery-type character while benefiting from enhanced surface reactivity.

The galvanostatic charge-discharge (GCD) profiles of the V_2_O_5_@PANI-NT and V_2_O_5_-NT electrodes were evaluated at current densities ranging from 0.5 to 2.0 A g^−1^ within a voltage window of 0.4–1.8 V (Figures 3D, 3E, and S13). The V_2_O_5_@PANI-NT-based cell delivered a specific capacity of 462.4 mAh g^−1^ at 0.5 A g^−1^, markedly surpassing that of the pristine V_2_O_5_-NT cell (352.3 mAh g^−1^) under identical conditions.

Electrochemical impedance spectroscopy (EIS) was conducted to elucidate the enhanced Zn^2+^ storage kinetics of the V_2_O_5_@PANI-NT composite. The Nyquist plots and corresponding equivalent circuit fitting (Figure S14A) reveal that the V_2_O_5_@PANI-NT exhibits a significantly lower internal resistance (*R*_*s*_ = 12.18 Ω) and charge-transfer resistance (*R*_*ct*_ = 25.19 Ω) than the V_2_O_5_-NT. This reduction indicates improved electrical conductivity and facilitated charge transfer at the electrode-electrolyte interface. Furthermore, the Zn-ion diffusion coefficient can be estimated based on the low-frequency Warburg region according to Equations 4, 5, and 6. As indicated in Figure S14B, the *σ*_*w*_ value of V_2_O_5_@PANI-NT was determined to be 63.75 Ω s^−0.5^, lower than that of V_2_O_5_-NT (73.24 Ω s^−0.5^). Accordingly, the Zn-ion diffusion coefficient of V_2_O_5_@PANI-NT was calculated as 8.71 × 10^−12^ cm^2^ s^−1^, which is higher than that of V_2_O_5_-NT(6.61 × 10^−12^ cm^2^ s^−1^), confirming accelerated Zn^2+^ diffusion in the composite. In addition, the relaxation time constant (τ_0_) derived from the Bode plot (Figure S14C) and Equation 4 decreased from 4.65 s for V_2_O_5_-NT to 1.21 s for V_2_O_5_@PANI-NT, reflecting a faster charging/discharging response. These collective EIS results demonstrate that the PANI modification effectively enhances the Zn^2+^ storage kinetics by enlarging the interlayer spacing, providing more active sites, and establishing efficient ion/electron transport pathways.

The long-term cycling stability was evaluated at a high current density of 2 A g^−1^. As shown in Figures 3F and S15, the V_2_O_5_@PANI-NT cell delivered a high discharge capacity of 238.2 mAh g^−1^ after an initial activation and retained 96.5% of its capacity over 500 cycles, with near-unity coulombic efficiency. In contrast, the V_2_O_5_-NT cell suffered from rapid capacity decay, retaining only 164.8 mAh g^−1^ (67.4% retention) after the same number of cycles. Post-cycling characterizations via SEM and EIS (Figure S16) confirmed the exceptional structural integrity of V_2_O_5_@PANI-NT, which exhibited minimal morphological change and maintained low charge transfer resistance, underscoring its superior electrochemical reversibility. This remarkable stability is attributed to the PANI coating, which effectively buffers structural strain and stabilizes the electrode-electrolyte interface. Furthermore, a comparative analysis with other recently reported cathodes (Table S1) highlights the competitive performance of the V_2_O_5_@PANI-NT composite.

In general, the excellent electrochemical performance of the V_2_O_5_@PANI-NT cell can be attributed to the following synergistic factors. (1) The hierarchical nanotube architecture offers a high specific surface area and porous structure, which not only shortens Zn^2+^ diffusion pathways but also provides abundant active sites, facilitating high specific capacity. (2) The intercalation of PANI serves as a structural pillar that effectively expands the V_2_O_5_ interlayer spacing, enhancing ionic mobility, while concurrently improving electronic conductivity, thus boosting both reaction kinetics and structural integrity. (3) The incorporation of PANI contributes additional Zn^2+^ storage sites and screens the strong electrostatic interactions between Zn^2+^ and the V_2_O_5_ host framework, effectively suppressing structural degradation and capacity fading during long-term cycling.

## Discussion

In summary, we demonstrate a novel and synergistic strategy for constructing high-performance V_2_O_5_-based cathodes for AZIBs by integrating nanotubular architectural engineering with PANI conductive polymer interfacial engineering. The unique core-shell nanotube morphology combined with PANI intercalation simultaneously addresses multiple challenges of V_2_O_5_ cathodes: the nanotube framework ensures mechanical stability and provides short ion diffusion paths, while PANI intercalation expands interlayer spacing, enhances electronic conductivity, and acts as a stable macromolecular pillar against structural degradation. The resulting PANI@V_2_O_5_-NT//Zn cell delivers a high specific capacity of 462.4 mAh g^−1^ at 0.5 A g^−1^ and exceptional cycling stability with 96.5% capacity retention after 500 cycles at 2.0 A g-^1^. This work provides not only a high-performance cathode material but also a general design principle for developing advanced electrode materials through the rational integration of morphological control and interfacial engineering.

### Limitations of the study

Despite the promising electrochemical performance achieved in this work, several limitations should be noted. First, the synthesis of V_2_O_5_ nanotubes relies on the use of 1-hexadecylamine as a structure-directing agent, which requires precise control over solvothermal conditions and subsequent calcination to remove organic residues. This multi-step process may pose challenges for large-scale and low-cost production. Second, while the PANI coating significantly enhances cycling stability, the underlying degradation mechanisms at the electrode-electrolyte interface during long-term cycling (beyond 500 cycles) remain to be fully elucidated. Third, due to equipment limitations, we were unable to perform *in situ* or operando structural characterizations (such as *in situ* XRD or TEM) to directly monitor the dynamic evolution of the core-shell structure during Zn^2+^ insertion/extraction. Such analyses could provide deeper insights into the structural stability and reaction kinetics of the composite material. Future work will focus on optimizing the synthesis scalability and employing advanced characterization techniques to further understand the interfacial behavior and long-term degradation pathways.

## Resource availability

### Lead contact

Further information and requests for resources and reagents should be directed to and will be fulfilled by the lead contact, Shen Wang (shenwang12@126.com).

### Materials availability

The V_2_O_5_ nanotubes and V_2_O_5_@PANI core-shell nanotube composites generated in this study have been synthesized in our laboratory following the procedures detailed in the STAR Methods section. All chemical reagents used are commercially available and can be sourced as described in the manuscript.

### Data and code availability

• All data generated and/or analyzed during this study are included in the article and supplementary figures and tables.
• This paper does not report the original code.
• Any additional information required to reanalyze the data reported in this paper is available from the lead contact upon request.

## Acknowledgments

The authors acknowledge support from 10.13039/501100016105Quzhou Science and Technology Assault Projects (2024K004), Scientific Research Startup Project of 10.13039/100020442Quzhou University (no. BSYJ202214), and 10.13039/501100012156Technical Project Entrustment (H2024165).

## Author contributions

S.W., conceptualization, formal analysis, investigation, methodology, writing – original draft, and writing – review and editing; X.S., data curation, formal analysis, and investigation; H.X., supervision and writing – review and editing.

## Declaration of interests

The authors declare no competing interests.

## STAR★Methods

### Key resources table

**Table:** 

REAGENT or RESOURCE	SOURCE	IDENTIFIER
**Chemicals, peptides, and recombinant proteins**		

Vanadium pentoxide	Aladdin	CAS: 1314-62-1
1-Hexadecylamine	Aladdin	CAS: 143-27-1
Aniline	Aladdin	CAS: 62-53-3
Hydrochloric acid	Aladdin	CAS: 7647-01-0
Ethanol AR, 99.5%	Sigma Aldrich	CAS: 64-17-5

**Software and algorithms**		

OriginLab	Analyze and graph	https://www.originlab.com/

**Other**		

Electrochemical workstation CHI 660E	electrochemical testing	http://www.chinstr.com/

### Experimental model and study participant details

There are no experimental models (animals, human subjects, plants, microbe strains, cell lines, primary cell cultures) used in the study.

### Method details

#### Preparation of V_2_O_5_ nanotube (V_2_O_5_ -NT)

First, 4 mmol of V_2_O_5_ was dispersed in 20 mL of ethanol and magnetically stirred for 10 min to form a homogeneous suspension. Subsequently, 4 mmol of 1-hexadecylamine was added, followed by continued magnetic stirring for 2 h. Then, 30 mL of deionized water and 10 mL of ethanol were added to the mixture, and stirring was maintained for 48 h to ensure sufficient intercalation of 1-hexadecylamine into the V_2_O_5_ layers. Afterwards, the mixture was sonicated for 30 min and transferred into a Teflon-lined autoclave for solvothermal reaction (180°C, 96 h). After the reaction, the product was washed several times with deionized water and ethanol, respectively. The washed sample was then dried in a vacuum oven at 60°C for 10 h. Finally, the as-prepared precursors were calcined at 300°C for 2 h under air atmosphere to obtain the V_2_O_5_ nanotube material.

In this study, V_2_O_5_-NT samples were systematically labeled according to synthesis parameters. Those synthesized with different molar ratios of 1-hexadecylamine to V_2_O_5_ are denoted as V_2_O_5_-NT-2/1, V_2_O_5_-NT-4/3, V_2_O_5_-NT-1/1, V_2_O_5_-NT-3/4, and V_2_O_5_-NT-1/2. Materials obtained under varying solvothermal reaction durations are designated as V_2_O_5_-NT-1, V_2_O_5_-NT-24, V_2_O_5_-NT-48, V_2_O_5_-NT-72, and V_2_O_5_-NT-96, where the numeric suffix indicates the reaction time in hours. Similarly, samples annealed at different temperatures are labeled as V_2_O_5_-NT-200, V_2_O_5_-NT-300, V_2_O_5_-NT-400, and V_2_O_5_-NT-500, corresponding to the thermal treatment temperature in degrees Celsius.

#### Synthesis of V_2_O_5_@PANI nanotube (V_2_O_5_@PANI-NT)

First, purified aniline was dissolved in water to prepare a 0.5 mol/L aqueous solution. Under ice-bath conditions with magnetic stirring, 3 M hydrochloric acid was added to adjust the pH to 2. Subsequently, the as-prepared V_2_O_5_-NT-300material (2 mol L^-1^) was added to the solution. Stirring was continued under ice-bath conditions for 1 h to facilitate the polymerization of aniline on the surface of the V_2_O_5_nanotubes, forming the V_2_O_5_@PANI composite material. The resulting product was then alternately washed three times with ethanol and deionized water. Finally, the obtained material was vacuum-dried at 60°C for 10 h to yield the V_2_O_5_@PANI nanotube composite.

In this study, the V_2_O_5_@PANI-NT samples were systematically labeled based on their synthesis parameters. Specifically, those synthesized under different pH conditions are designated as V_2_O_5_@PANI-NT-0.5, V_2_O_5_@PANI-NT-1, V_2_O_5_@PANI-NT-1.5, V_2_O_5_@PANI-NT-2, and V_2_O_5_@PANI-NT-2.5, respectively.

#### Fabrication of quasi-solid-state zinc-ion batteries

The cathode slurry was prepared by mixing active material (V_2_O_5_@PANI-NT or V_2_O_5_-NT), Ketjen black, and polytetrafluoroethylene (PTFE) binder at a mass ratio of 8:1:1 in ethanol. The homogeneous slurry was uniformly cast onto functionalized carbon cloth and dried at 60°C overnight to obtain free-standing electrodes.

For zinc-ion battery assembly, Zn foil served as the anode and ZnSO_4_/PVA gel as the solid-state electrolyte. The gel electrolyte was synthesized by dissolving 4 g ZnSO_4_ and 5 g PVA in 30 mL deionized water at 85°C under continuous stirring for 4 h. Both electrodes (4 cm × 4 cm) were immersed in the gel electrolyte for 5 min, then hot-pressed together and air-dried for 24 h to form a quasi-solid-state zinc-ion battery.

#### Characterization and measurements

Structural and compositional characterization was performed as follows: X-ray diffraction (XRD) analysis employed a Bruker D8 Advance diffractometer with Cu Kα radiation (λ = 1.5418 Å). Thermal gravimetric profiles were acquired in ambient atmosphere using a Shimadzu DTG-60H instrument (20–800°C temperature range, 5°C min^-1^ heating rate). Specific surface area and pore-size distribution were determined via Brunauer-Emmett-Teller (BET) methodology on an ASAP 2020 analyzer. Microstructural evaluation utilized field-emission scanning electron microscopy (FE-SEM; ZEISS SUPRA 55) and high-resolution transmission electron microscopy (HRTEM; Tecnai G2 F30, 200 kV). Chemical states and elemental composition were probed by X-ray photoelectron spectroscopy (XPS; Thermo Scientific ESCAlab 250Xi spectrometer).

#### Electrochemical measurements

For electrochemical testing, galvanostatic charge-discharge (GCD), cyclic voltammetry (CV), and electrochemical impedance spectroscopy (EIS, 0.01∼100 kHz with an amplitude of 5 mV) were performed using a CHI 760f electrochemical workstation. The cycling stability was evaluated by Neware CE-6000 battery testing system. All experiments were conducted at room temperature (25°C).

The peak current *(i*) and scan rate (*v*) obey the following linear relationship using Equation 1:^30^^,^^31^

(Equation 1) $$i=av^{b}$$

(Equation 2) log(i)=blog(v)+log(a)

The b-value is a slope calculated from Equation 2 that reveals whether the electrochemical reaction is dominated by surface capacitance-controlled or diffusion-controlled processes. A b = 1 indicates that the charge storage process is controlled by the surface redoxreaction (capacitive contribution), whereas a b of 0.5 indicates that the charge storage is controlled by ion diffusion.^20^^,^^21^

Besides, the surface capacity (*k*_*1*_*v*) and diffusion-controlled (*k*_*2*_*v*^*0.5*^) contribution can be calculated based on the following Equation 3:^18^^,^^23^^,^^31^

(Equation 3) $$i=k_{1}v+k_{2}v^{0.5}$$

The Zn ion diffusion coefficient can be estimated according to the Equations 4, 5, and 6:^4^^,^^5^^,^^6^^,^^12^^,^^14^^,^^16^

(Equation 4) $$D_{{\text{Zn}}^{2+}}=\frac{R^{2}{\;T}^{2}}{2{A^{2}\;n^{4}\;F^{4}\;C^{2}\;\sigma }^{2}}$$

(Equation 5) $$Z^{\prime }=R_{1}+R_{2}+\sigma \omega^{-0.5}$$

(Equation 6) $$\omega^{-0.5}={\left(2\pi f\right)}^{-0.5}$$

In this equation, $D_{{Zn}^{2+}}$ denotes the diffusion coefficient of Zn^2+^ ions (cm^2^ s^-1^), *R* is the gas constant (8.314 J mol^-1^ K^-1^), *T* is the absolute temperature(K), *A* is the surface area of electrode (cm^2^), *F* refers to Faraday constant (96485 C mol^-1^), *C* is the molar concentration of Zn^2+^ in the electrolyte (mol cm^-3^), and σ_w_ is Warburg impedance coefficient (Ω s^-0.5^).

The relaxation time constant (τ_0_) of the electrode is defined as the reciprocal of the characteristic frequency (ƒ_0_) at a phase angle of -45° in the Bode plot (τ_0_ = 1/ƒ_0_). It characterizes the transition point between the dominant capacitive behavior (at frequencies below ƒ_0_) and the resistive behavior (at frequencies above ƒ_0_). The calculation formula is as shown in Equation 7:^11^^,^^16^^,^^25^

(Equation 7) $$\tau_{o}=\frac{1}{f_{o}}$$

### Quantification and statistical analysis

No methods were used to determine whether the data met the assumptions of the statistical approach.

### Additional resources

Our study has not generated or contributed to a new website/forum or has not been part of a clinical trial.
