# Supplementary material for: Interface engineering V2O5@PANI nanotube for high-performance aqueous zinc-ion batteries
Source: iScience. 2025 Dec 4;29(1):114337. doi: 10.1016/j.isci.2025.114337 (PMC12774708; doi:10.1016/j.isci.2025.114337)
Supplement: Document S1. Figures S1–S16 and Table S1 [file mmc1.pdf]

**iScience, Volume *29***

## Supplemental information

# Interface engineering $V_2O_5$@PANI nanotube for high-performance aqueous zinc-ion batteries

**Shen Wang, Xueying Sun, and Hongbo Xu**

# Supporting Information

## Interface Engineering $V_2O_5$@PANI Nanotube for High-performance Aqueous Zinc-Ion Batteries

Shen Wang,[1*] Xueying Sun,[2] Hongbo Xu[1*]

[1] China College of Chemical and Material Engineering, Quzhou University, 324000, Quzhou, China

[2] MIIT Key Laboratory of Critical Materials Technology for New Energy Conversion and Storage, School of Chemistry and Chemical Engineering, Harbin Institute of Technology,150001, Harbin, China.

***Correspondence: shenwang12@126.com, iamxhb@hit.edu.cn**

Reaction time exerts a pronounced influence on the morphology of the resulting products. To investigate the formation process of $V_2O_5$ nanotubes, a series of comparative experiments were conducted by varying the solvothermal reaction duration. Given that the self-rolling transition from sheet-like to tube-like structures occurs under solvothermal conditions, the morphologies of samples obtained at different reaction times were systematically characterized, as shown in Figure S1.

SEM images reveal a distinct morphological evolution with increasing reaction time. Under high-temperature conditions, the layers of the lamellar material initially undergo delamination and curling, a behavior attributed to stress disparities resulting from unsaturated bonds between adjacent layers. As the reaction proceeds, the exfoliated layers gradually self-roll and ultimately form uniaxially oriented $V_2O_5$ nanotubes. With prolonged solvothermal treatment, the nanotube walls undergo gradual fusion and further structural refinement, eventually yielding well-defined uniaxial nanotubes with enhanced structural integrity.

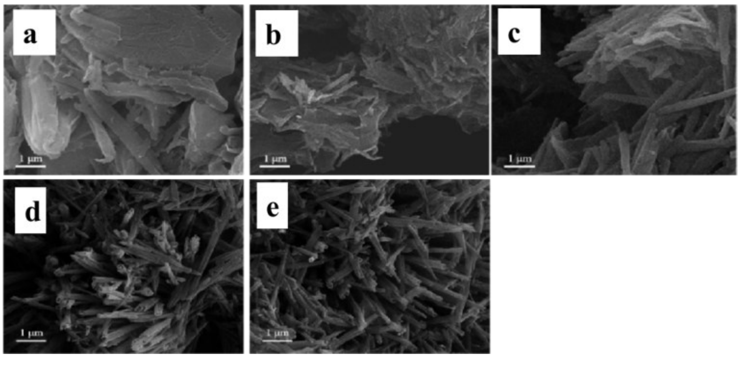


Figure S1. SEM images of $V_2O_5$-NT percussor with different solvothermal treatment time:(a) $V_2O_5$-NT-1, (b) $V_2O_5$-NT-24, (c) $V_2O_5$-NT-48, (d) $V_2O_5$-NT-72, (e) $V_2O_5$-NT-96

To elucidate the structural evolution during the formation of $V_2O_5$ nanotubes, X-ray diffraction (XRD) analysis was performed on samples obtained at various reaction times (Figure S2). Comparison of the XRD patterns revealed that the diffraction peaks of the precursor material matched well with the standard PDF reference. Following intercalation, the characteristic peaks corresponding to the (200), (001), and (101) crystal planes of layered $V_2O_5$ shifted toward lower angles, indicating an expansion of the interlayer spacing. In the early stages of the solvothermal reaction, these peaks continued to shift to lower angles, confirming further enlargement of the interlayer distance—consistent with the initial curling of sheet-like structures observed by SEM.

After 24 hours of reaction, the (001) peak began to shift toward higher angles, suggesting a gradual contraction of the interlayer spacing. This stage corresponds to the uniaxial scrolling and fusion of the layered sheets into tubular architectures. The decrease in interlayer distance is likely due to the partial extrusion of 1-hexadecylamine molecules from the interlamellar spaces during the scrolling process, resulting in a more compact nanotube structure.

Combined with the time-dependent morphological evolution illustrated in the SEM images (Figure S1), the optimal reaction time for forming well-defined $V_2O_5$ nanotubes was determined to be 96 hours. Accordingly, all subsequent experiments in this study were carried out under this optimized condition.

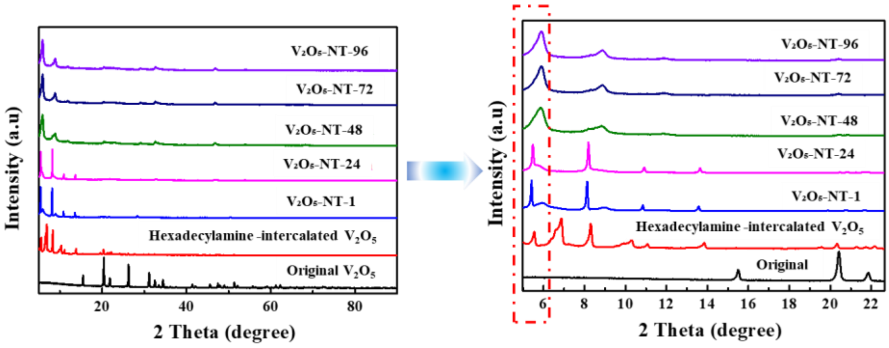


Figure S2. XRD patterns of $V_2O_5$-NT percussor with different solvothermal treatment time

Figure S3(a) illustrates the formation mechanism of $V_2O_5$ nanotubes, which involves multiple stages. Initially, 1-hexadecylamine undergoes protonation in aqueous solution, producing alkylammonium ions and hydroxide ions, thereby establishing a mildly alkaline environment. Under these conditions, $V_2O_5$ reacts with $OH^-$ ions,

resulting in the cleavage of V–O bonds and the formation of $VO_3^-$ ions. Subsequently, positively charged alkylammonium ions intercalate into the interlayers of $VO_3^-$ via electrostatic interactions, promoting their self-assembly into an ordered lamellar structure. The edges of this layered configuration contain unsaturated bonds, leading to localized energy instability. Additionally, the cleavage of V–O bonds generates $V^{4+}$–O and $V^{5+}$ species with distinct ionic radii. Under solvothermal conditions, partial reduction of $V^{5+}$ to $V^{4+}$ occurs, inducing internal stress within the layered framework. This stress drives the bending of the layered sheets, which ultimately undergo uniaxial scrolling to form nanotube structures, thereby effectively mitigating the energy instability caused by unsaturated edge bonds. Throughout this morphological evolution, the material transitions successively from initial particles (Figure S3(b)) to an intercalated intermediate (Figure S3(c)), and finally, under solvothermal treatment, forms nanotubes via uniaxial rolling (Figure S3(d)).

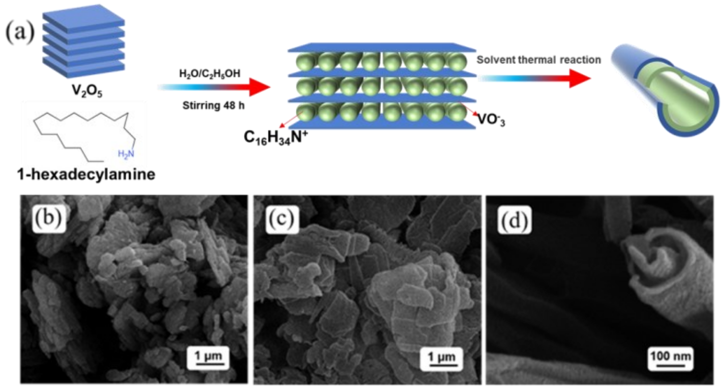


Figure S3. (a) Schematic diagram of the formation mechanism of $V_2O_5$-NT, (b) original $V_2O_5$ particles, (c) Hexadecylamine-intercalated $V_2O_5$, (d) $V_2O_5$-NT obtained via solvothermal reaction

The solvothermally synthesized $V_2O_5$-NT samples retain residual organic impurities, such as 1-hexadecylamine, which served as a structure-directing agent during synthesis. These residues remain embedded within the nanotube framework and may act as insulating barriers, impeding ion transport and reducing accessible electroactive sites, thereby adversely affecting the capacity and rate performance in energy storage devices such as batteries and supercapacitors. Thus, calcination is necessary to remove organic residues and optimize the electrochemical properties of the material.

SEM images(Figure S4 a-e) revealed progressive morphological changes with increasing annealing temperature: the nanotube walls gradually thickened and contracted, consistent with the removal of intercalated hexadecylamine that initially provided structural support. Upon annealing at 400 °C, the nanotubes underwent structural collapse, forming dense aggregates. This degradation is attributed to the complete decomposition of organic templates, which eliminated interlayer support, coupled with enhanced crystallinity and internal stress within the $V_2O_5$ lattice at elevated temperatures.

XRD analysis further elucidated the microstructural evolution. With increasing temperature, the (00l) diffraction peaks shifted toward higher angles, reflecting a continuous decrease in interlayer spacing due to the removal of hexadecylamine. After annealing at 400 °C or above(Figure S4f), the XRD pattern closely matched that of standard bulk $V_2O_5$ (PDF#41-1426), indicating restoration of the pristine layered structure and complete elimination of organic species. These results confirm the critical role of hexadecylamine in stabilizing the nanotubular morphology.

In summary, while annealing effectively removes organic residues, excessive temperatures (>400 °C) induce structural collapse due to loss of interlayer support and increased crystallinity-related stress. Optimizing thermal treatment conditions is essential to balancing impurity removal with retention of the nanotubular architecture for enhanced electrochemical performance.

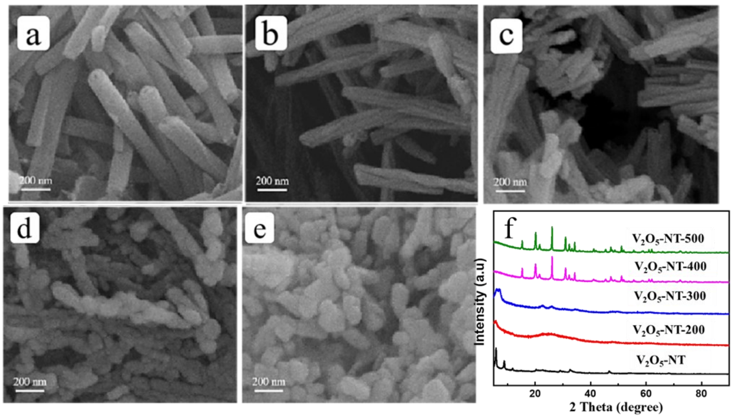


Figure S4. SEM images(a-e) and XRD patterns of $V_2O_5$-NT with different thermal treatment temperature: (a) $V_2O_5$-NT, (b) $V_2O_5$-NT-200, (c) $V_2O_5$-NT-300, (d) $V_2O_5$-NT-400 , (e) $V_2O_5$-NT-500

To directly observe microstructural changes, $V_2O_5$-NT samples before and after annealing at 300 °C were examined by TEM (Figure S5). The interlayer spacing in both

samples exhibited certain distributional variations, consistent with XRD analysis. These differences can be attributed to varying arrangements of intercalated alkyl chains, such as overlapping or divergent tilt angles. TEM images confirmed that both samples retained an open-ended tubular morphology. High-resolution imaging revealed a periodic alternation of VOx layers (dark contrast) and hexadecylamine molecular layers (light contrast) within the nanotube walls. The average nanotube diameter decreased markedly from approximately 200 nm before annealing to 100–110 nm after annealing. HRTEM measurements further indicated a reduction in interlayer spacing from 2.14 nm to 0.98 nm following thermal treatment. This contraction is primarily due to the removal of most hexadecylamine intercalant, resulting in the loss of interlayer support and overall structural densification. These findings align with prior hypotheses, confirming that annealing effectively eliminates organic intercalants and induces significant structural contraction in $V_2O_5$ nanotubes.

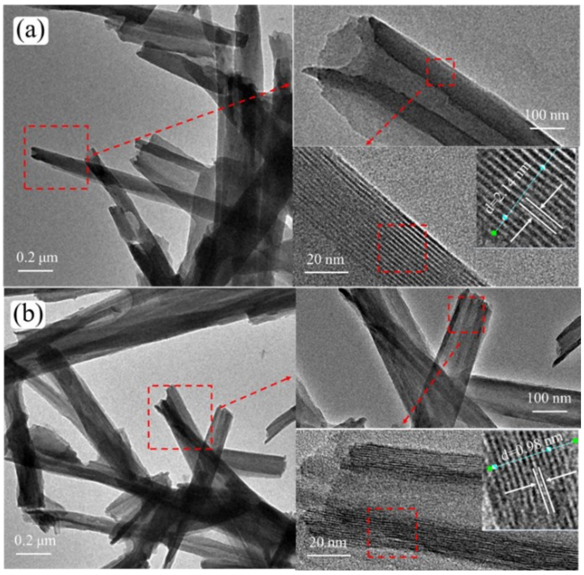


Figure S5. TEM image and HRTEM image: (a) $V_2O_5$-NT-300 precursor and (b) $V_2O_5$-NT-300 .

The survey spectra (Figure S6a, c) confirm the presence of O, C, N, and V in all samples. The C and N signals originate from the hexadecylamine intercalant. After annealing, a pronounced decrease in the intensities of the C 1s and N 1s peaks was observed, indicating substantial removal of C and N—consistent with the thermal decomposition of organic species. Moreover, the main V peak shifted from 514 eV to 516 eV, suggesting an increase in the average vanadium valence state following annealing[1-3].

High-resolution V 2p spectra are presented in Figures S6b and S6d. For the pristine sample ($V_2O_5$-NT), the V $2p_{1/2}$ peak of $V^{4+}$ was located at 522.1 eV, while the V $2p_{3/2}$ region exhibited deconvoluted peaks at 514.7 eV ($V^{4+}$) and 516.04 eV ($V^{5+}$), with a $V^{4+}/V^{5+}$ peak area ratio of approximately 2:1[4-6].After annealing at 300 °C ($V_2O_5$-NT-300), the $V^{4+}$ components shifted to 523.1 eV (V $2p_{1/2}$) and 515.2 eV (V $2p_{3/2}$), and the $V^{5+}$ peak appeared at 516.3 eV. The $V^{4+}/V^{5+}$ ratio reversed to approximately 1:2.

These results clearly indicate an increase in the binding energy of the V $2p_{3/2}$ orbital and a higher proportion of $V^{5+}$ species in the annealed sample, confirming an elevated average vanadium valence state. In electrochemical contexts, a higher vanadium valence state (e.g., $V^{5+}$) is conducive to enhanced ion intercalation capacity and improved electrode performance, owing to its richer redox activity and stronger electrostatic attraction for ions.

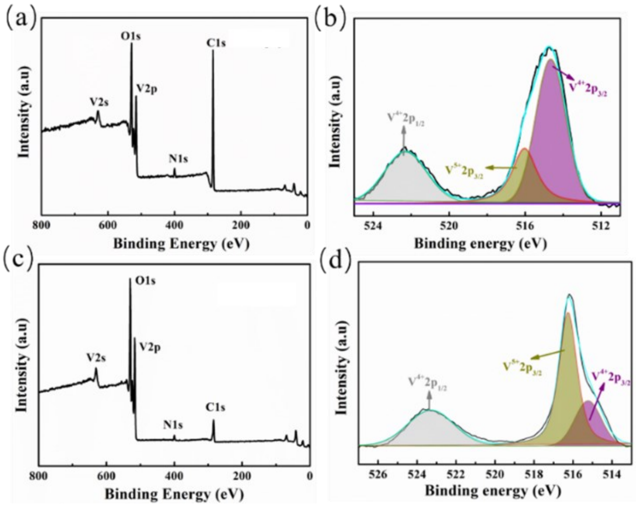


Figure S6. X-ray photoelectron spectroscopy spectra: $V_2O_5$-NT-300 precursor (a) survey spectrum and (b) Narrow-scan XPS spectra of V2p; $V_2O_5$-NT-300(a) survey spectrum and (b) Narrow-scan XPS spectra of V2p

The pH of the solution is a key parameter governing the polymerization rate of aniline. To systematically evaluate the influence of pH on the morphology of the resulting composites, this study employed a controlled-variable approach to characterize the reaction behavior across different pH conditions, with the aim of identifying the optimal synthesis parameters. Under acidic conditions with ice-bath stirring, aniline monomers intercalate into the interlayer gaps of $V_2O_5$ nanotubes and undergo in situ polymerization driven by the strong oxidizing capacity of $V_2O_5$,

ultimately forming core–shell structured $V_2O_5$@PANI composites.

pH profoundly modulates the in situ polymerization kinetics of polyaniline, particularly during the chain initiation step involving anilinium cations. As shown in the SEM results in Figure S7, lower pH values (i.e., stronger acidity) correspond to faster polymerization rates and more pronounced etching of the $V_2O_5$ nanotubes. At pH 0.5 and 1, the reaction proceeds too aggressively, compromising the integrity of the nanotube morphology. In contrast, at pH 2.5, the polymerization rate is markedly slow, leading to inadequate polyaniline deposition on the $V_2O_5$ surface and thus diminished composite performance. Through comprehensive morphological and structural comparisons across pH conditions, pH 2 was identified as optimal, yielding $V_2O_5$@PANI-NT-2 composites that retain well-defined nanotube architectures and exhibit promising potential for zinc-ion storage applications.

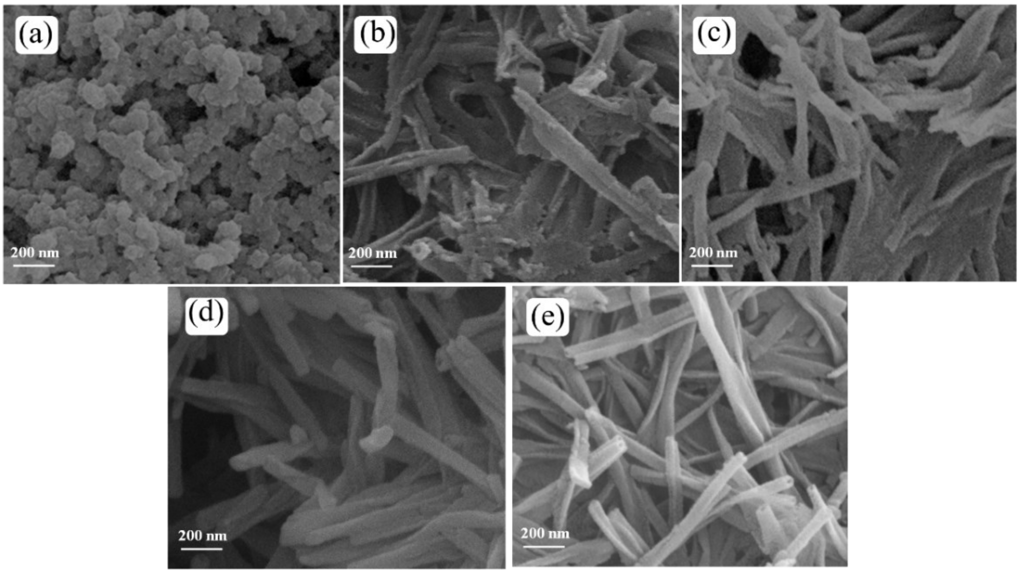


Figure S7. SEM images of the products under different pH conditions:(a) $V_2O_5$@PANI-NT-0.5, (b) $V_2O_5$@PANI-NT-1, (c) $V_2O_5$@PANI-NT-1.5, (d) $V_2O_5$@PANI-NT-2, and (e) $V_2O_5$@PANI-NT-2.5

The physicochemical properties of $V_2O_5$@PANI composites prepared by aniline and $V_2O_5$ nanotubes at different reaction times (1 h, 2 h, 3 h, and 4 h) were investigated in this study at pH=2. As can be observed from the SEM images in Figure S8, the polymerization reaction of aniline continued with the reaction time, and the walls of the $V_2O_5$ nanotubes were gradually etched while the content of polyaniline attached to their surfaces was increasing. However, when the reaction time exceeded 3 h, it was difficult

to maintain the original tubular structure due to the excessive etching of the $V_2O_5$ nanotubes, and at the same time, the excessively generated polyaniline might undergo cross-linking and agglomeration.

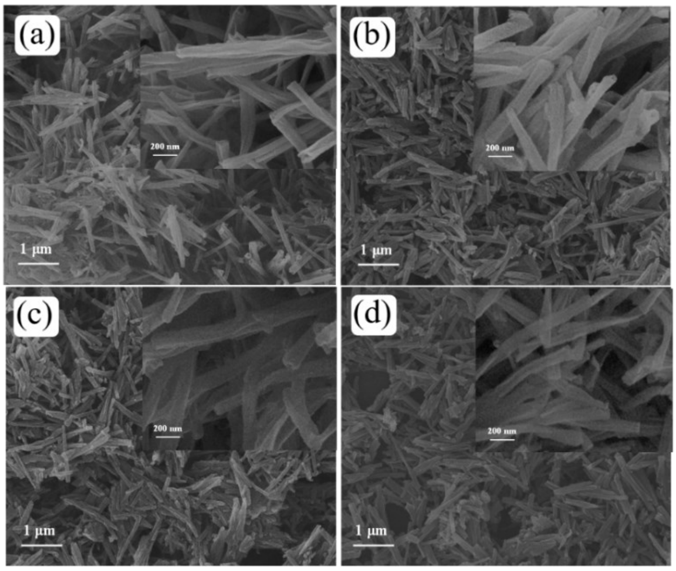


Figure S8. SEM images of the $V_2O_5$@PANI-NT-2 under different reaction time：(a) 1 h，(b) 2 h，(c) 3 h，(d) 4 h

XRD characterization of the $V_2O_5$@PANI composites synthesized at different reaction times (Fig. S9) offers insights into their structural evolution. With prolonged reaction time, the intensity of the characteristic $V_2O_5$ diffraction peaks gradually diminished, accompanied by the emergence of a broad amorphous halo in the 20°–30° range, attributable to polyaniline. This suggests successful incorporation and progressive accumulation of polyaniline within the composite. Further analysis of the XRD patterns reveals a continuous shift of the (001) diffraction peak toward lower angles, indicating intercalation of polyaniline molecules into the interlayer galleries of $V_2O_5$. This expansion of interlayer spacing confirms adaptive structural rearrangements within the $V_2O_5$ crystal lattice during composite formation.

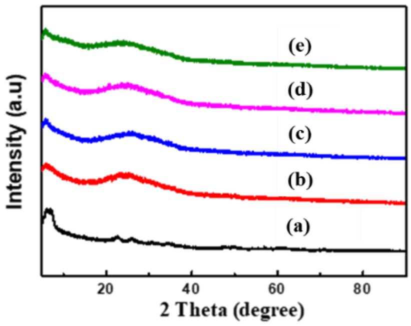


Figure S9. XRD patterns of the $V_2O_5$@PANI-NT-2 under different reaction time: (a) 0 h, (b) 1 h, (c) 2 h, (d) 3 h, (e) 4 h

Fig. 10a displays the Raman spectra of $V_2O_5$@PANI-NT and $V_2O_5$-NT. The characteristic vibrations of $V_2O_5$ appear as peaks below 1100 $cm^{-1}$. The stretching vibration of O–(V)$_3$, V–O–V and V=O bonds are identified at 664 cm-1, 812 $cm^{-1}$ and 989 $cm^{-1}$, respectively. After the synthesis process, new peaks emerging in the range of 1100 $cm^{-1}$ to 1700 $cm^{-1}$ correspond to the stretching modes of PANI. The peaks at 1387 $cm^{-1}$ and 1650 $cm^{-1}$ are attributed to the D (defect/disorder) and G (graphitic) bands of carbon materials, respectively. In-plane C–H bending vibrations are represented by the peak at 1182 $cm^{-1}$. The peaks at 1493 $cm^{-1}$ and 1339 $cm^{-1}$ are associated with the C=N stretching mode of the quinone group and the stretching modes of C–N bonds, respectively.[3, 7-11] Multiple peaks within the 1100–1700 $cm^{-1}$ range collectively confirm the presence of benzene-ring-derived vibrational modes, consistent with the successful incorporation of PANI[5].

Fourier transform infrared spectroscopy (FT-IR) confirmed the successful formation of the PANI cladding. As shown in Fig. S10b, the FTIR spectrum of PANI-$V_2O_5$-NT exhibits several characteristic bands in addition to those of $V_2O_5$-NT: a peak near 1605 $cm^{-1}$ corresponding to the stretching and bending vibrations of the N–H bond. The band at 1305 $cm^{-1}$ is attributed to the C-N stretching vibration within the benzene ring. Moreover, the peak near 1240 $cm^{-1}$ arises from the C-N stretching mode of the PANI backbone. Peaks observed at 1580 $cm^{-1}$, 1305 $cm^{-1}$ and 1140 $cm^{-1}$ are assigned to the stretching and bending vibrations of the aromatic ring C = C, C–C bond and C–H bond, respectively. The presence of these additional absorption peaks in the PANI-$V_2O_5$-NT sample (red line), which are absent in the $V_2O_5$-NT spectrum (black line),

provides clear evidence for the incorporation of PANI. In addtion, the bands between 11100 $cm^{-1}$ to 500 $cm^{-1}$ are associated with the symmetric and asymmetric vibrations of V–O vibrations. The peak at approximately 830 $cm^{-1}$ indicates the characteristic absorption of V–O–V bridging bonds. Notably, the shift of the V=O stretching vibration from 1011 $cm^{-1}$ in $V_2O_5$-NT to 985 $cm^{-1}$ in PANI-$V_2O_5$ suggests partial reduction of $V^{5+}$ to $V^{4+}$ cations[8, 12-14].

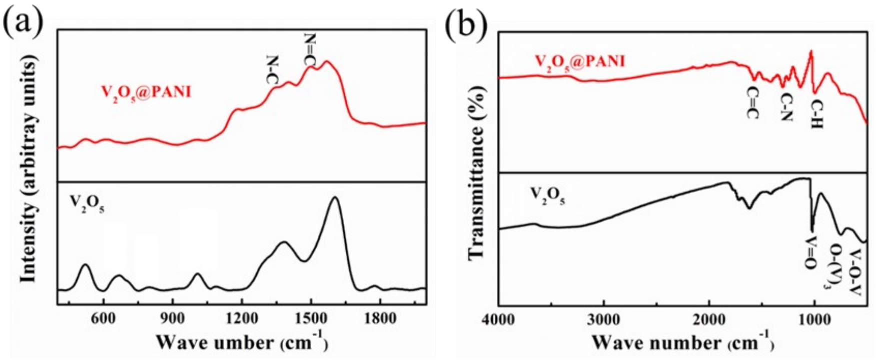


Figure S10. (a) Raman spectra and (b) FT-IR of $V_2O_5$@PANI-NT and $V_2O_5$ -NT

The presence of water and polyaniline (PANI) molecules within the $V_2O_5$@PANI-NT composite was further confirmed by thermogravimetric (TG) analysis. As shown in Fig. S11, distinct differences are observed between the samples before and after PANI incorporation. The PANI-$V_2O_5$-NT composite exhibits a weight loss of approximately 5.8 wt.% in the same temperature range, which is attributed to the release of intercalated water. Furthermore, a weight loss of about 23.4 wt.% occurs between 200 and 400 °C, corresponding to the decomposition of PANI[3, 15, 16].

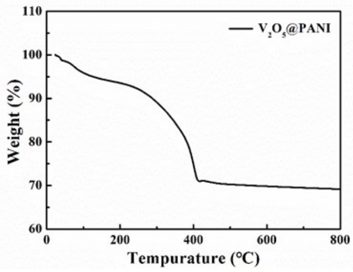


Figure S11. TG profiles of $V_2O_5$@PANI-NT

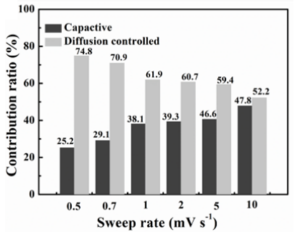


Figure S12. Capacitive and diffusion contributions of $V_2O_5$ -NT

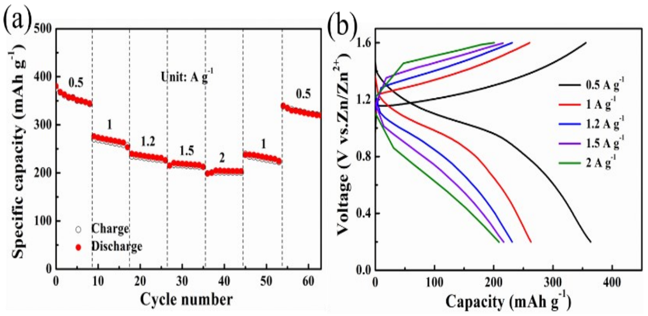


Figure S13. (a) Rate performance and (b) Charge- discharge curves of $V_2O_5$ -NT at different current densities

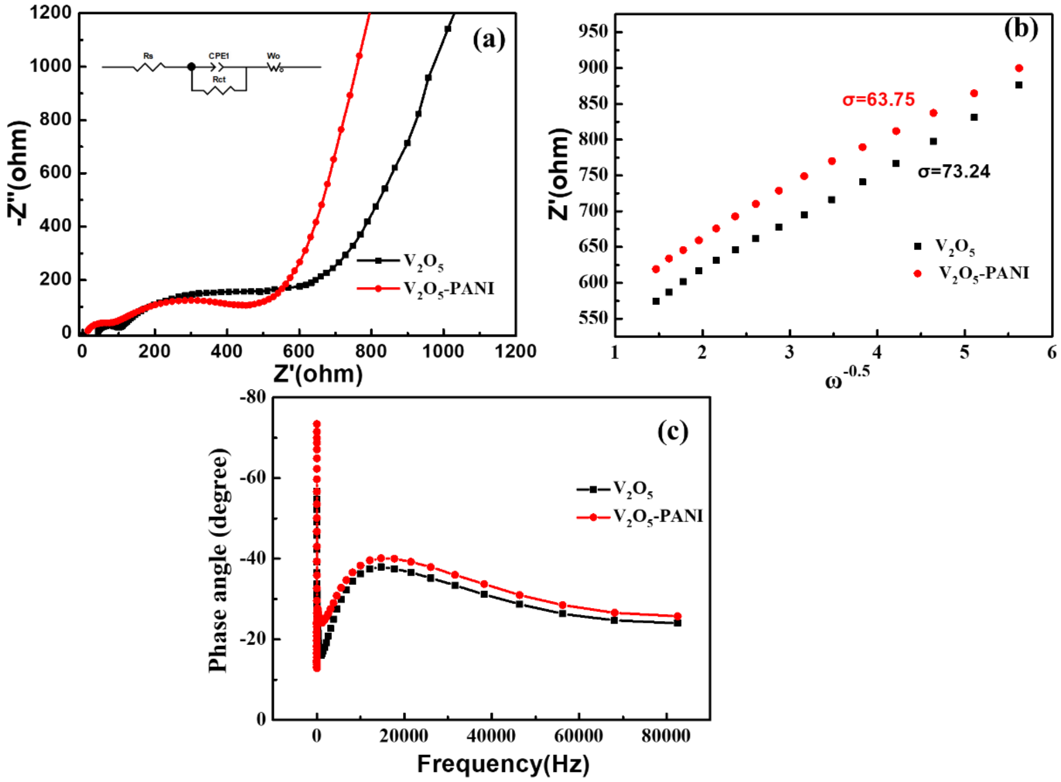


Figure S14. Electrochemical impedance analysis of $V_2O_5$-NT and $V_2O_5$@PANI-NT electrodes: (a) Nyquist plots, (b) The linear relationship between *Z'* and $\omega^{-0.5}$ in the low frequency region, and (c) Bode plot of phase angle and frequency

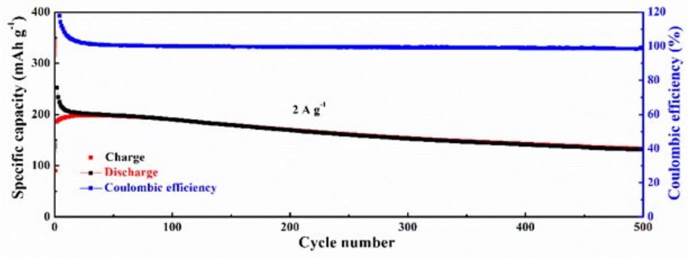


Figure S15. Cycling performance of $V_2O_5$ -NT at 2.0 A g$^{-1}$

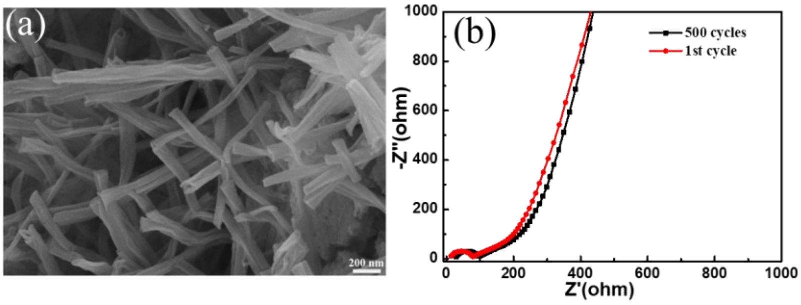


Figure S16. (a) SEM and (b) Nyquist plots of $V_2O_5$@PANI-NT after 500cycles

**Table S1.** Comparison on the electrochemical performance of some Zn-ion batteries.

| Cathode | Capacity /mAh g$^{-1}$ | Rates /A g$^{-1}$ | Cycle numbers | Ref |
|---|---|---|---|---|
| $NH_4$-$V_2O_5$ | 310.8 | 0.1 | 1000 | 12 |
| $Na_{0.76}V_6O_{15}$@PANI | 400.5 | 0.1 | 5000 | 10 |
| PANI/$V_2O_5$ | 384.0 | 0.1 | 1000 | 1 |
| $Mg^{2+}$-doped $V_3O_7H_2O$ | 382.7 | 0.1 | 2500 | 13 |
| ZnHCF/PANI | 130.0 | 0.2 | 350 | 8 |
| $VOPO_4$/graphene | 168.4 | 0.1 | 1000 | 14 |
| $V_2O_5$ hollow nanotubes | 283.4 | 0.1 | 50 | 15 |
| $V_2O_5$ $nH_2O/Ti_3C_2T_x$ MXene | 323.0 | 0.1 | 50 | 16 |
| Flower-spherical $Cu_{0.4}V_2O_5$ | 267.0 | 0.1 | 1000 | 17 |
| Co-doped $V_2O_5$ | 437 | 0.2 | 1400 | 18 |
| Poly(catechol)-Intercalated Vanadium Oxide | 466.4 | 0.1 | 1100 | 19 |
| **$V_2O_5$@PANI-NT** | **462.4** | **0.5** | **500** | **This work** |

## References


1. Y. Zhu, X. Liu, X. Hu, T. Wang, I. P. Parkin, M. Wang and B. D. Boruah, *Chemical Engineering Journal*, 2024, **487**, 150384.
2. M. S. Javed, H. Lei, Z. Wang, B.-t. Liu, X. Cai and W. Mai, *Nano Energy*, 2020, **70**, 104573.
3. W. Bi, J. Wang, E. P. Jahrman, G. T. Seidler, G. Gao, G. Wu and G. Cao, *Small*, 2019, **15**, 1901747.
4. H. Wang, M. Liang, H. Ma, C. Ma, W. Duan, H. Yang, Z. He, Y. Zhao and Z. Miao, *Journal of Energy Storage*, 2024, **101**, 113785.
5. R. Roy, P. Sharma, G. Mahendra, P. Dutta, V. Raghuraman, P. Singh and A. K. Singh, *Advanced*

*Energy Materials*, 2025, e02262.
6. J. Luo, M. Cao, N. Naresh, J. Borah, S. Li, T. Wang, B. K. Sarma, J. Yao, I. P. Parkin and B. D. Boruah, *Advanced Functional Materials*, 2025, **35**, 2417607.
7. Y. Zhang, Z. Li, B. Zhao, Z. Wang and J. Liu, *Journal of Materials Chemistry A*, 2024, **12**, 1725-1735.
8. Q. Liu, Z. Ma, Z. Chen, M. Cui, H. Lei, J. Wang, J. Fei, N. He, Y. Liu and Q. Liu, *Chemical Communications*, 2022, **58**, 8226-8229.
9. M. Lira-Cantú and P. Gomez-Romero, *Journal of Solid State Chemistry*, 1999, **147**, 601-608.
10. R. Jia, C. Yin, B. Wang, L. Li and J. Hu, *Chemical Engineering Journal*, 2024, **500**, 157587.
11. X. Wang, Y. Wang, J. Hao, Y. Liu, H. Xiao, Y. Ma, L. Chen, Y. Huang and G. Yuan, *Energy Storage Materials*, 2022, **50**, 454-463.
12. Y. Lu, T. Wang, N. Naresh, J. Borowiec, I. P. Parkin and B. D. Boruah, *Nano Research Energy*, 2024, **3**, e9120125.
13. Y. Shi, B. Yin, Y. Sun, R. Ge, Y. Hu, J. Li, H. Li, S. Zhang and T. Ma, *Chemical Communications*, 2024, **60**, 8597-8600.
14. J. Jiang, Y. Huang, Z. Fan, Y. Cui, X. Liu and X. Wang, *Chemical Communications*, 2025, **61**, 6190-6193.
15. X. Liang, G. Gao, S. Feng, Y. Du and G. Wu, *Journal of Alloys and Compounds*, 2019, **772**, 429-437.
16. G. Xu, Y. Zhang, Z. Gong, T. Lu and L. Pan, *Journal of Colloid and Interface Science*, 2021, **593**, 417-423.
17. H.-J. Zeng, J.-T. Huang, Y.-B. Chen, L. Chen, Z.-H. Wu, H.-Y. Yang and Z. Chen, *Tungsten*, 2025, 1-10.
18. Q. Li, N. Yu, L. Li, B. Sun, X. Chen, F. Wei, Q. Wang, Y. Sui, J. He and Z. Zhang, *Journal of Power Sources*, 2025, **628**, 235895.
19. Z. Li, Y. Zhang, L. Zhang, J. Li, Z. Wang, X. Wang, K. Ding, H. Liu and Y. Wang, *ACS Applied Energy Materials*, 2025, **8**, 5442-5454.
